# Attitudes towards sexuality and related caregiver support of people with intellectual disabilities: A systematic review on the perspectives of people with intellectual disabilities

**DOI:** 10.1111/jar.12928

**Published:** 2021-07-08

**Authors:** Wouter de Wit, Wietske M. W. J. van Oorsouw, Petri J. C. M. Embregts

**Affiliations:** ^1^ Tranzo, Tilburg School of Social and Behavioral Sciences Tilburg University Tilburg The Netherlands; ^2^ Zuidwester Middelharnis The Netherlands

**Keywords:** attitudes, intellectual disabilities, sex education, sexual health, sexuality, support

## Abstract

**Background:**

Sexual health remains at risk for people with an intellectual disability. Attitudes towards sexuality, its support and education have an important role in promoting sexual health. The current review aims to provide an overview of the current research on supportive and restrictive attitudes towards sexuality and its support of people with intellectual disabilities themselves.

**Method:**

A systematic review was conducted, searching across eight databases. The quality of the studies was assessed with the Mixed‐Method Appraisal Tool.

**Results:**

Six themes emerged from the data: sexual behaviour, sexual identity, intimate relationships, barriers to sexual expression, sex education and support by caregivers. Supportive and restrictive attitudes were reported throughout.

**Conclusions:**

Attitudes regarding sexuality of people with intellectual disabilities are heterogeneous and people with intellectual disabilities seem to be able to express their sexual desires, needs and attitudes. Findings allow for improved individual support and in‐depth research questions.

## 1 INTRODUCTION

The sexual health of people with intellectual disabilities has been a subject of interest since the emergence of the normalisation movement in the 1970s (Ailey et al., 2003; McGuire & Bayley, 2011). In general, sexuality and sexual health are viewed as accessible to everyone, irrespective of disabilities (Ailey et al., 2003). Sexual health refers to the freedom to express one's sexuality in an enjoyable manner, free from disease, coercion and abuse (WAS, 2014). For one's sexual health to flourish, people must be afforded the possibility to experience sexuality in all its diversity, including sex, gender identities and roles, sexual orientation, eroticism, pleasure, intimacy and reproduction (WHO, 2015).

Sexual health has proven to be an elusive goal for some people with intellectual disabilities (Sinclair et al., 2015) because their access to sexual experiences can be restricted, and their knowledge and skills limited (Schaafsma et al., 2014; Servais, 2006). Concerning sexual experiences, people with intellectual disabilities are less likely to have had sexual relations than those without disabilities (Baines et al., 2018; Gil‐Llario et al., 2018; Kijak, 2013). Furthermore, unsafe sex occurs more frequently, as does victimisation from sexual abuse (Baines et al., 2018; Gil‐Llario et al., 2018). Regarding knowledge, people with intellectual disabilities are known to have various misconceptions about sexuality topics, such as the physical changes that transpire during puberty (Kijak, 2011), reproduction and sexual transmitted disease (STDs; Jahoda & Pownall, 2014). Furthermore, people with intellectual disabilities often lack relevant skills, for example, dating, maintaining intimate relationships (Brown & McCann, 2018; Kijak, 2011) and sexual decision‐making (McGuire & Bayley, 2011). The combination of less positive sexual experiences, knowledge and skills has led to the conclusion that sexual health remains at risk for people with intellectual disabilities (AAIDD, 2008).

In conjunction with sexual experiences, knowledge and skills, attitudes concerning the sexuality of people with intellectual disabilities are considered a contributing factor to the promotion of their sexual health (Ailey et al., 2003; Travers et al., 2014). *Attitudes* refer to thoughts, emotions and feelings concerning a certain subject (Prislin & Crano, 2008)—for example, people's thoughts or opinions towards homosexuality, and their beliefs or emotions about the right for people with intellectual disabilities to have sexual relations. Supportive attitudes on sexuality appear to have a positive impact on people's sexual health (e.g., Ford et al., 2019), as restrictive attitudes can have a negative effect (Dionne & Dupras, 2014; McCann et al., 2019; Servais, 2006). Until recently, research on sexuality‐related attitudes among people with intellectual disabilities has had a predominant focus on the existence and meaning of restrictive attitudes, often phrased as barriers towards sexuality (Servais, 2006; Sinclair et al., 2015). Such studies showed, among other things, that people with intellectual disabilities can hold restrictive attitudes towards sexual expressions such as the belief that sexual relations were not allowed for them (Dionne & Dupras, 2014; English et al., 2018). Recently, a broader focus on sexuality‐related attitudes has emerged in literature, including both restrictive and supportive attitudes (Brown & McCann, 2018; McGuire & Bayley, 2011). However, a comprehensive review of the broad range of attitudes of people with intellectual disabilities regarding their own sexuality is currently lacking. Ultimately, such an overview could provide new entry points for promoting sexual health of people with intellectual disabilities.

In the present systematic literature review that is reported in accordance with the Preferred Reporting Items for Systematic Reviews and Meta‐Analysis (PRISMA) statement (Liberati et al., 2009), the attitudes of people with intellectual disabilities concerning the broad scope of their own sexuality (i.e., according to the working definition of the WHO, 2015) were identified and analysed. During the selection process, both supportive and restrictive attitudes were included. For people with intellectual disabilities, support and education are integral for their possibilities of expressing their sexuality (Brown & McCann, 2018; Schaafsma et al., 2015). Therefore, the attitudes among people with intellectual disabilities towards sexuality‐related support (i.e., provided by support staff and family) were also included. As the current review aims to provide in‐depth insight into the attitudes among people with intellectual disabilities, only studies underpinned by a qualitative design were selected.

## 2 METHOD

### 2.1 Search strategy

To be as exhaustive as possible in identifying relevant studies, a search strategy was build based on the Exhaustive Search Method (ESM) (Bramer et al., 2018). As a result, the search was carried out within seven databases (i.e., Embase, Medline Ovid, Web of Science, Cochrane Central, PsychINFO Ovid, CINAHL EBSCOhost and Google Scholar) and built through a single‐line‐search strategy that was based on free text search terms. The search strategy was optimised through adding relevant search terms using the thesauruses of the databases and comparing the relevance of these results. The authors were supported by an information specialist, with expertise in ESM.

In addition to ESM, the Population, Intervention/exposure, Control and Outcome (PICO) approach (Liberati et al., 2009), was used to identify relevant keywords and synonyms in order to form the search terms. The Population component was *Adults with intellectual disabilities*. Examples of relevant population‐related search terms were as follows: ‘intellectual disability’, ‘developmental disabilities’ and ‘learning disabilities’. The Intervention/exposure component concerned the *Sexuality of people with intellectual disabilities*. Sexuality content had to refer to at least one dimension of sexuality formulated in the working definition of the World Health Organisation (WHO, 2015), that is, sex, gender identities and roles, sexual orientation, eroticism, pleasure, intimacy and reproduction. Studies focusing exclusively on sexual risks (e.g., sexual abuse, unwanted pregnancy, STDs, parenthood for people with intellectual disabilities) were excluded. Examples of search terms were: ‘sex’, ‘sexual’, ‘homosexual’, ‘love’, ‘romance’, ‘marriage’, ‘masturbation’ and ‘intercourse’. The PICO‐Control component was not applicable in this review because of the descriptive nature of our research question. The Outcome component concerned *Attitudes*, which encompassed cognitive, affective and behavioural intentions (Prislin & Crano, 2008), and can be of an explicit or implicit nature (Bassili & Brown, 2005). Examples of search terms were as follows: ‘attitude’, ‘value’, ‘norm’, ‘view’, ‘opinion’ and ‘experience’.

Relevant search terms were listed for each PICO component (i.e., adults with intellectual disabilities; sexuality of people with intellectual disabilities and attitudes) based on MeSH terms and keywords, utilising synonyms, subcategories and singular, plural and verbal forms. All components were combined with the Boolean operator ‘AND’, and synonyms were divided by ‘OR’. See Table 1 for an example of the search in Embase.

**TABLE 1:** Search terms and synonyms Embase^a^

*Population*	
People with intellectual disabilities	intellectual impairment OR mental deficiency OR mentally disabled person OR learning disorder OR developmental disorder OR intell* OR mental* OR cognit* OR neurocognit* OR impair* OR disab* OR handicap* OR deficien* OR retard* OR deficit* OR disabilit* OR limitation* OR idioc* OR retard* OR down syndrome* OR development* disab* OR development* delay* OR development* disorder* OR learning* disab*
Direct care professionals	professional* OR personnel* OR staff OR provider* OR nurse* OR nursing OR worker* OR attendant* OR field‐worker* OR fieldworker* OR residential‐care* OR care‐giver* OR caregiver* OR carer*
Family carers	famil* OR parent* OR father* OR mother* OR sibling* OR brother* OR sister* OR relatives OR first‐degree‐relative*
*Intervention/exposure*	
Sexuality	gender identity OR sexuality OR intersex OR sex worker OR love OR birth control OR sexual desire OR marriage OR menstrual cycle OR penis erection OR sexual education OR contraceptive device OR sexualit* affectivit* OR intimate‐relationship* OR transgender* OR bicurious OR bisexual* OR cross‐sex* OR crossgender* OR female‐to‐male OR gay OR gays OR gender‐variant OR intersex* OR pleasure* OR contact* OR physical* OR reproduct* OR behav* OR protect* OR responsib* OR counsel* OR fantas* OR desire OR longing OR relation* OR interact* OR anal OR oral OR experien* OR career* OR activit* OR satisf* OR body‐part OR body‐image OR anatom* OR educat* OR needs OR favour* OR OR marriage OR family‐life educat* OR masturbat* OR blowjob OR cybersex* OR genderqueer* OR homosexual* OR intersex* OR lesbian* OR transexual* OR transgender* OR transvestit* OR intercourse* OR erotic* OR auto‐erotic* OR promisc* OR courtship* OR dating OR libido heterosexualit* OR prostit* OR pornograph* OR escort‐service* OR escortservice* OR intimac* OR love OR romance OR coitus OR penetrat* OR ‘birth control’ OR contracept* OR sterilizat* OR the‐pill OR condom OR family‐planning OR menstrual OR menstruat* OR erection* OR orgasm*
*Outcome*	
Attitude	attitude OR preference OR satisfaction OR social norm OR social stigma OR prejudice OR taboo OR value* OR discriminat* OR judgement* OR criticism* OR considerat* OR reasoning OR perspective* OR thought* OR thinking OR knowledge* OR affect OR affection OR emotion* OR feeling* OR like OR liking OR dislike OR disliking OR favour OR disfavour OR opinion* OR decision OR judge OR experience* OR virtue* OR reflect* OR view OR views OR impression* OR aware* OR reali* OR belief* OR instinct* OR marginali* OR neglect* OR ignor* OR supportive OR filiation*

^a^ Similar search strategies were used for Medline Ovid, CINAHL, Psych INFO, Web of Science, Cochrane CENTRAL and Google Scholar, with adjustments to the search terms based on the applicable thesaurus and MeSH terms.

A systematic literature search was conducted for original, peer‐reviewed articles published in English between January 1997 and June 2020. The search originally consisted of a broad search for studies examining the attitudes among people with intellectual disabilities, their support staff and family caregivers. The present study is limited to studies investigating the attitudes among people with intellectual disabilities themselves.

### 2.2 Study selection

**FIGURE 1:**
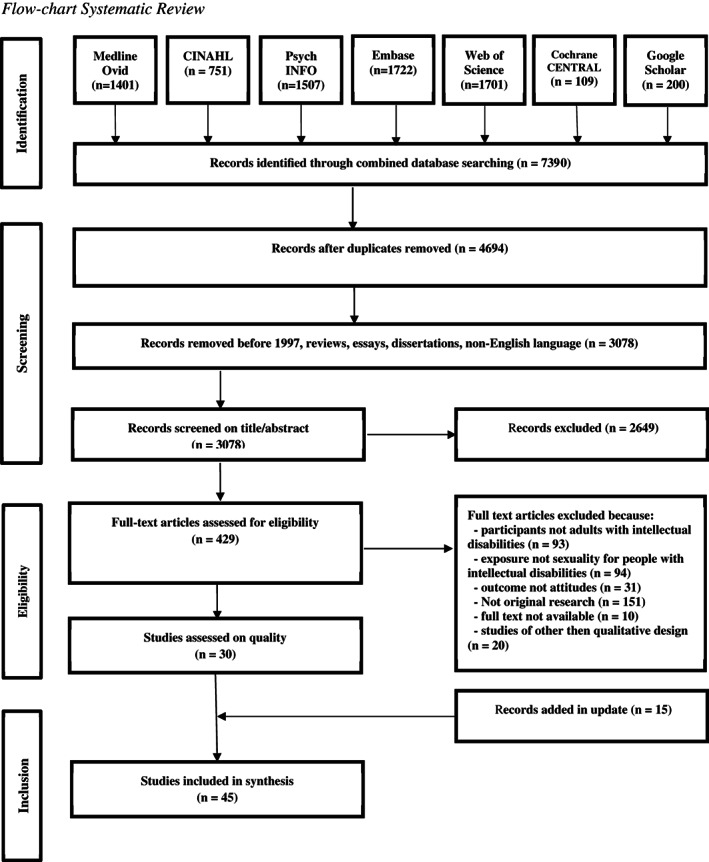
Flowchart systematic review

Studies were selected along four consecutive phases: (1) identification, (2) screening, (3) eligibility and (4) selection (see Figure 1; Moher et al., 2009). To guide the study selection, inclusion and exclusion criteria were formulated for each PICO component. See Table 2 for a complete overview of inclusion and exclusion criteria. In the first identification phase, databases were searched using a predefined search string, which resulted in 7390 records. Subsequently, duplicates, reviews, essays and dissertations were removed in the screening phase. The first and second author independently screened 3038 articles on title. Based on predetermined inclusion and exclusion criteria (see Table 2), both authors agreed on 81% of all study titles. In the case of disagreements, titles were then discussed with the third author until full consensus was reached. Following the screening on title, the abstracts of the 1499 remaining articles were screened in relation to the inclusion and exclusion criteria by two independent authors (i.e., first and second author). The abstract screening results in an inter‐rater agreement of 73%. Again, abstracts were discussed until full consensus was reached, while the third author was consulted for complex cases.

**TABLE 2:** Inclusion and exclusion criteria

*Inclusion criteria*
Population: Adult people with intellectual disabilities. Participants reported with IQ rates of 80 or below OR participants (when IQ was not specified) more generally described as ‘people with intellectual disability’ or ‘people with learning disability’. AND Ages 18–65 years old OR when ages were not specified, the population was described as ‘adults’, ‘(wo)men’ or ‘people’. Exposure: Studies concerning the sexuality of adult people with intellectual disabilities. Outcome: Outcomes concerning the attitudes or affiliations of attitudes (e.g., views) and can be cognitive, affective or behavioural. General: Peer‐review journals. Study design was qualitative or mixed methods.
*Exclusion criteria*
Population People with cognitive impairments other than an intellectual disability or having an illness or disorder associated with intellectual disability, for example, epilepsy or autism. The participant sample consists of sexual offenders or victims of sexual abuse. Exposure Studies concerning negative sexual experiences (e.g., sexual abuse, offending). Studies concerning the production or provision of pornography, prostitution and social‐erotic services (Nb. Studies concerning the *use* of these services were included). Studies concerning STDs. Studies concerning reproduction. Studies concerning parenting. Outcome Outcomes concerning attitudes of the general population, relatives and/or professionals. Outcomes concerning actual experiences (Nb. Subjective evaluations of these experiences were included). General: Studies not presenting original research data. Studies on psychometric data (i.e., validity and reliability of measures). Studies dating before 1997. Studies published in other language than English.

Abbreviation: IQ, intelligence quotient; STD, sexual transmitted disease.

Third, in the eligibility phase, the full texts of the remaining articles (*n* = 419) were screened in relation to the inclusion and exclusion criteria in three successive steps, namely (1) population (i.e., adults with intellectual disabilities), (2) exposure (i.e., sexual health) and (3) outcome (i.e., attitudes). Each full text selection step was conducted by the first author and thoroughly discussed with the second author until full consensus was reached. Again, the third author was consulted in complex cases. The remaining articles (*n* = 68) were critically appraised using an instrument suitable for the assessment of various research designs, namely the Mixed‐Method Appraisal Tool, version 2018 (Hong et al., 2018). All the articles were appraised by the first author and extensively discussed with the second author, until full agreement was achieved. Lastly, after removing all articles that either included family and support staff or were underpinned by a quantitative study design, 30 articles were included in the selection phase for data extraction and analysis.

### 2.3 Data extraction and analysis

Our final selection consisted of qualitative studies, which employed a broad range of research methods (e.g., focus groups, thematic analysis, Interpretative Phenomenological Approach). The authors opted for a synthesis method that would enable the standardised scrupulous processing of this large range of qualitative data. Therefore, a meta‐synthesis was conducted, which comprised five consecutive steps (Lachal et al., 2017). First, all the texts were carefully read and reread, until a thorough comprehension of the content was ascertained. Second, data were extracted via line‐by‐line coding. Third, the codes were grouped and categorised into a hierarchical tree structure. Codes with similar meanings were grouped together and an overarching category was administered to the created subtheme. For example, codes involving cuddling, kissing and intercourse were categorised under the subtheme entitled ‘Attitudes towards sexual behaviour’. Finally, overarching analytical themes were generated by comparing and discussing all subthemes within the research group. All final themes were discussed and agreed upon by the three reviewers. An update of the search was carried out on 23 June 2020. Fifteen articles were added following the aforementioned procedure, resulting in a final selection of 45 articles. Data were confirmed and no new themes were added.

## 3 RESULTS

The final selection included 45 studies, 42 of which were qualitative designs and three were mixed‐method studies, of which only the qualitative data were included. Ten studies were conducted in UK, four in Australia, The Netherlands and USA; three in Canada, Ireland and Sweden; two in Finland, Malta and Taiwan and one each in Belgium, China, Croatia, Iceland, Israel, Poland, South Africa and Spain. Of the 913 participants in total, 306 were labelled as having mild intellectual disabilities, 24 moderate intellectual disabilities and 32 severe intellectual disabilities. The severity of intellectual disability was not specified for 551 participants. The participants included 425 males, 453 females and five participants who preferred another gender typology, while for 30 participants in the original studies, gender was not specified. Data extraction and analyses the following six themes: sexual behaviour, sexual identity, intimate relationships, barriers to sexual expression, sex education and support by caregivers (see Table 3). Each theme contained multiple subthemes and main codes. The content of each theme is elaborated upon below.

**TABLE 3:** Overview themes reported in studies

Study	Sexual behaviour	Sexual identity	Intimate relationships	Barriers	Sex education	Support
Abbott and Burns (2007)		X	X	X	X	X
Azzopardi Lane et al. (2019)	X		X	X		X
Bane et al. (2012)	X	X	X	X		X
Bates et al. (2017)			X			
Bernert and Ogletree (2013)	X		X	X	X	
Björnsdóttir et al. (2017)		X	X	X		X
Buljevac et al. (2020)			X			X
Callus et al. (2019)			X			
Chou et al. (2008)			X			
Chou et al. (2015)	X	X	X	X	X	X
Darragh et al. (2017)	X					
Dinwoodie et al. (2016)		X	X	X		X
Fitzgerald and Withers (2013)	X	X		X	X	X
Healy et al. (2009)	X		X	X		X
Johnson et al. (2002)	X		X			
Kelly et al. (2009)	X		X	X	X	X
Kijak (2013)	X			X		
Lesseliers and Hove (2002)	X		X	X		X
Löfgren‐Mårtenson (2008)	X		X	X		
Löfgren‐Mårtenson (2009)	X					
Löfgren‐Mårtenson (2012)				X	X	X
Mattila, Maatta, and Uusiautti (2017)			X			
Mattila, Uusiautti, and Maatta (2017)			X			
McClelland et al. (2012)	X		X	X		
Muswera and Kasiram (2019)	X		X	X		
Neuman (2020)			X			X
Oakes and Thorpe (2019)	X				X	X
O'Shea and Frawley (2020)	X		X			X
Pariseau‐Legault and Holmes (2017)	X					
Rojas et al. (2016)	X		X	X		X
Rushbrooke et al. (2014)	X	X	X	X		X
Schaafsma et al. (2017))	X		X	X	X	X
Scior (2003)			X	X		
Scott et al. (2014)			X			
Sitter et al. (2019)	X		X	X	X	X
Stoffelen et al. (2013)	X	X	X	X	X	X
Stoffelen et al. (2018)	X	X	X	X		X
Stoffelen et al. (2019)	X			X	X	X
Turner and Crane (2016a)	X		X	X		X
Turner and Crane (2016b)	X		X	X	X	X
Walmsley et al. (2016)	X					X
Wheeler (2007)	X		X			X
Wilkinson et al. (2015)			X	X		
Yacoub and Hall (2009)	X		X	X		X
Yau et al. (2009)	X	X	X	X		
*Total*	*31*	*10*	*36*	*29*	*12*	*27*

### 3.1 Theme One: Attitudes towards sexual behaviour

Thirty‐one studies reported on participants' attitudes towards sexual behaviour. Two subthemes were identified as follows: (1) potential reasons for engaging in sexual behaviour and (2) the perception of different aspects and forms of sexual behaviour.

First, five studies reported on the attitudes towards potential reasons for engaging in sexual behaviour, which concerned: (1) gender–role expectations (e.g., ‘*I know you have sex with a man ‘cos you're a woman*’; Fitzgerald & Withers, 2013, p. 7), (2) play (e.g., ‘*I just did it to play*…’; Lesseliers & Hove, 2002, p. 74) and (3) a desire to have children (Azzopardi Lane et al., 2019; Bernert & Ogletree, 2013).

Second, attitudes pertaining to the perception of sexual behaviour were reported in 31 studies. Participants with intellectual disabilities expressed both supportive and restrictive views towards various forms of social‐sexual and auto‐erotic behaviour. Concerning social‐sexual behaviour, participants were quoted on their perception of a range of behaviours, including holding hands, kissing, cuddling, touching, touching of each other's genitals and sexual intercourse (e.g., Bernert & Ogletree, 2013; Chou et al., 2015; Lesseliers & Hove, 2002; Pariseau‐Legault & Holmes, 2017; Stoffelen et al., 2019). For instance, ‘*I love it –we…just lay together, kiss and cuddle – fondle*’ (Turner & Crane, 2016a, p. 684). Participants often used rather generic expressions such as ‘*Sex is pleasure for two*’ (Schaafsma et al., 2017, p. 28). Some participants clearly viewed sex as something nice and desirable, for example, stating that ‘*I like to touch and kiss*’ (Turner & Crane, 2016a, p. 688). Other participants viewed sex as no fun, yucky or disgusting; as one participant opined: ‘*It's disgustingly gross*.’ (Bernert & Ogletree, 2013, p. 244).

For some participants, their perception of social‐sexual behaviour was associated with the issue of consent; for example, ‘*If a girl don't want sex, it's no good having sex with her*’ (Healy et al., 2009, p. 909). For others, it was condom use that influenced their perception. For example, one man reported that condoms should always be used (Stoffelen et al., 2013, p. 261), whereas others expressed a preference for not wearing condoms ‘*Cause it feels better without*’ (Bernert & Ogletree, 2013; Schaafsma et al., 2017). However, some participants associated contraception more with averting menstruation and pain, than with sexual expression (Muswera & Kasiram, 2019).

Attitudes regarding auto‐erotic behaviour were reported less frequently (*n* = 9) in comparison to social‐sexual behaviour (*n* = 23) and pertained to masturbation (Lesseliers & Hove, 2002; Pariseau‐Legault & Holmes, 2017) and watching pornography (Chou et al., 2015; Darragh et al., 2017; Löfgren‐Mårtenson, 2008; Rushbrooke et al., 2014). Masturbation was referred to in a somewhat excusatory manner (i.e., ‘*I only do that [masturbation] at night, I like to play but not always*’; Lesseliers & Hove, 2002, p. 76), as part of a broader questioning about whether masturbation was part of sexuality (‘*I have [sex] toys and I don't know if that's part of it*’; Pariseau‐Legault & Holmes, 2017, p. 608) or viewed as unhealthy behaviour (Chou et al., 2015). However, one woman expressed that she felt relaxed after masturbation, and that it helped her to learn how ‘… *to make love with my boyfriend someday*.’ (Pariseau‐Legault & Holmes, 2017, p. 608). Furthermore, attitudes towards looking at erotic pictures on the Internet were reported, as participants with intellectual disabilities viewed this as an acceptable form of behaviour (Löfgren‐Mårtenson, 2008).

### 3.2 Theme Two: Attitudes towards sexual identity

Ten studies presented attitudes of participants with intellectual disabilities towards sexual identity. Two different subthemes were addressed, reflecting (1) gender identity and (2) attitudes towards Lesbian, Gay, Bisexual or Transgender (LGBT) identities. First, participants reflected on their general beliefs about gender identity (i.e., what it means to be a man or a woman). For instance, asking someone on a date ‘*… is up to the boy*’ (Bane et al., 2012, p. 117), men are considered more courageous (Chou et al., 2015), a man needs to have a job and he should not drink or smoke (Yau et al., 2009). Women were characterised as needing to be tender and generous (Yau et al., 2009). In addition, for some, being a woman was associated with pain from labour and menstruation (Fitzgerald & Withers, 2013).

Secondly, participants discussed the meaning of having an LGBT identity in six studies. Some participants believed it was possible to tell if someone was gay, for example, ‘*because the way they dress*’ (Dinwoodie et al., 2016, p. 6). Others said ‘*You cannot see that I am bisexual*.’ (Stoffelen et al., 2018, p. 257). On a related note, a broad range of views towards accepting LGBT identities were reported. Some participants were struggling with their own LGBT identity (‘*I thought there was something wrong with me*’; Dinwoodie et al., 2016, p. 7; ‘*Hard and just, no, it [exploring sexual identity] was just hard I think, yeah. A bit weird as well*’ Rushbrooke et al., 2014, p. 535). Other participants talked of complete acceptance and expressed: ‘*I am proud to be gay*’ (Stoffelen et al., 2013, p. 261).

### 3.3 Theme Three: Attitudes concerning intimate relationships

Attitudes of participants with intellectual disabilities towards intimate relationships (i.e., being boyfriend and girlfriend) were stated in almost 80% of the included studies (*n* = 36). The reported attitudes reflected (1) reasons for engaging in a relationship and (2) expectations from a relationship, and the characteristics participants were looking for in a partner.

Attitudes on the reasons for engaging in a relationship were reported in 29 studies. A recurring attitude in these studies was that having a relationship was a goal in and of itself (e.g., Bates et al., 2017; Johnson et al., 2002; Stoffelen et al., 2013; Yau et al., 2009). In addition, participants viewed a relationship as a possible way to have sex (Turner & Crane, 2016b; Yau et al., 2009). Others believed that relationships bring positive feelings, for instance, ‘*I like having girlfriends. They make me feel good*’ (Healy et al., 2009, p. 908). Other participants were seeking company; ‘*And that's why I want to get a girlfriend, because I don't want to be stuck on my own*’ (Rushbrooke et al., 2014, p. 534). Similarly, some participants sought security in their lives. This was often related to their preference of finding a non‐disabled partner, or, as one participant noted: ‘*if he is normal, he can afford to take care of me*’ (Yau et al., 2009, p. 103). Furthermore, a relationship was desired to increase their sense of autonomy over their life. For example, one participant expressed ‘*then, I would be master in my own house*’ (Lesseliers & Hove, 2002, p. 77). Besides the wealth of reasons cited for engaging in an intimate relationship, there were participants with intellectual disabilities who expressed a strong desire to remain single. As one man stated: ‘*Not interested in women anymore, they just ruin your life*’ (Yacoub & Hall, 2009, p. 8).

Alongside attitudes towards reasons for engaging in relationships, 28 articles presented quotes in which participants described their attitudes towards both what to expect from a relationship, and the desired attributes they were looking for in a relationship partner. First, participants referred to the physical appearance of their partner (e.g., Bates et al., 2017; Bernert & Ogletree, 2013; Kelly et al., 2009; Neuman, 2020; Turner & Crane, 2016a). For instance, one participant explained: ‘*His eyes drive me crazy. He has blue eyes and I just look into them. It's like I see his soul or something. I love his eyes, his eyes, his chest. And muscular. Duane's muscular right here*.’ (Turner & Crane, 2016a, p. 685). Another participant explained how medical aids acted as a turn‐off: ‘*Went to her bedroom and a breathing mask and I thought “no thanks”*.’ (Bates et al., 2017, p. 608). Next, participants pointed to the importance of (small) outings with their partner, like walking together (Bane et al., 2012) or going to the movies (Abbott & Burns, 2007). In addition to this, having someone to share aspects of daily life with (e.g., having someone to talk to, share everyday worries with) was deeply appreciated (e.g., Schaafsma et al., 2017; Turner & Crane, 2016b; Yau et al., 2009). Other participants emphasised the importance of sharing similar interests (Mattila, Maatta, & Uusiautti, 2017; Mattila, Uusiautti, & Maatta, 2017). As a counterpart to this, some participants expressed the need for time apart, for example, ‘*It's impossible [to be together] 24 hours a day; you need space*’ (Neuman, 2020, p. 137).

Further elaborating on the attitudes of participants with intellectual disabilities towards intimate relationships and/or romantic partners, participants also emphasised how one should interact. First, romance was viewed as an important aspect within relationships (Bane et al., 2012; Kelly et al., 2009), which included things like teasing (Turner & Crane, 2016a) and buying Valentines Day gifts (Kelly et al., 2009). Participants also stressed the importance of helping and caring: ‘*What's being in love? Caring for somebody*,….’ (Abbott & Burns, 2007, p. 32) and ‘*I want to*… *look after her. And, take care of her*’ (Turner & Crane, 2016b, p. 2307). Conversely, some participants explained the importance of being helped and cared for by their partner: ‘*It's good to have a boyfriend because they*… *mind you and help you and stuff like that*’ (Bane et al., 2012, p. 117). As one participant outlined, helping and caring works in both directions: ‘*Well, I want someone to love and care for me, and I want to care for them as well. It works two ways*.’ (Abbott & Burns, 2007, p. 32). The reciprocal nature of relationships was also mentioned in extracts concerning trust and respect. For instance, ‘*It's important to treat each other well*….’ (Bane et al., 2012, p. 116) and ‘… *take it easy and get to know each other well*.’ (Lesseliers & Hove, 2002, p. 73). Furthermore, some participants were of the attitude that partners should be ‘… *friendly, courteous, and kind*’ (Turner & Crane, 2016b, p. 2309). Lastly, it was reported that having the possibility of relationships cannot go without the possibility of breaking up (Sitter et al., 2019).

### 3.4 Theme Four: Attitudes related to barriers to sexual expression

Participants with intellectual disabilities frequently expressed attitudes concerning the barriers they encountered when pursuing their sexual expression (*n* = 29). The reported views on barriers can roughly be divided into (1) barriers outside of the participants' influence and (2) barriers related to their individual characteristics.

With respect to barriers outside of the participants' control, three types of barriers were reported across 14 different articles. First, some participants experienced a lack of privacy needed for intimate contact due to the fact that they lived with others (i.e., living with their parents or in an institution; e.g., Healy et al., 2009; Kelly et al., 2009; Muswera & Kasiram, 2019; Rushbrooke et al., 2014). For some participants, this resulted in looking for alternative locations (i.e., primarily outdoors, in public places, bathhouses), even though these places were considered to be unsafe and uncomfortable (McClelland et al., 2012). Second, participants who felt ready to get married indicated barriers such as a wedding being too expensive for them (Lesseliers & Hove, 2002), first needing a steady income (Scior, 2003) and the necessity of (first) securing proper housing (Yau et al., 2009). Third, participants referred to the availability of potential partners, ranging from a belief that finding a partner was difficult (Dinwoodie et al., 2016; Löfgren‐Mårtenson, 2012) to experiencing an overabundance of potential partners (Rojas et al., 2016; Rushbrooke et al., 2014). Furthermore, participants with LGBT identities believed that finding a partner was even more difficult for them (Dinwoodie et al., 2016; Stoffelen et al., 2013). Some participants mentioned successfully overcoming these difficulties through engaging in online dating, using dating services in newspapers or seeking help from dating coaches. However, others stated it was better ‘*not to have too high expectations*’ (Löfgren‐Mårtenson, 2008, p. 131).

Participants also reported four types of barriers in 21 articles, which pertained to individual characteristics. First, having an intellectual disability was viewed as a barrier (Abbott & Burns, 2007; Healy et al., 2009; Rojas et al., 2016; Schaafsma et al., 2017). Second, barriers occurred in the form of embarrassment associated with either asking someone out on a date (Bane et al., 2012; Chou et al., 2015; Rushbrooke et al., 2014) or talking about their innermost desires with their partners (Lesseliers & Hove, 2002; Turner & Crane, 2016b; Yacoub & Hall, 2009). Furthermore, some participants added that they did not even know how to date (Chou et al., 2015; Stoffelen et al., 2018). A third and recurring subtheme concerned participants' worries about how support staff and their families would react to them having a sexual relationship. For example, some feared being removed from the institution, the day centre or even their parents' home (e.g., Fitzgerald & Withers, 2013; Kelly et al., 2009; Stoffelen et al., 2013; Turner & Crane, 2016a). Lastly, some participants abstained from engaging in sexual activities due to worries about STDs and unwanted pregnancies (Bernert & Ogletree, 2013; Björnsdóttir et al., 2017; Fitzgerald & Withers, 2013; Rushbrooke et al., 2014; Yau et al., 2009).

### 3.5 Theme Five: Attitudes towards sex education

Twelve studies reported attitudes towards sex education, referring to (1) the perception of sex education in general, (2) the content of sex education and (3) the features that contribute to successful sex education. In five studies, sex education was appreciated by participants as being an important subject (Löfgren‐Mårtenson, 2012; Oakes & Thorpe, 2019; Schaafsma et al., 2017; Stoffelen et al., 2013). Conversely, other participants perceived some aspects of sex education as embarrassing (e.g., discussing masturbation: Chou et al., 2015; ‘… *put on condom on a fake cock*’; Löfgren‐Mårtenson, 2012, p. 215) or frightening (e.g., watching childbirth films: Löfgren‐Mårtenson, 2012). Some studies reported participants' outspoken views towards the content of the provided education. They believed that sex education should address more than simply pregnancy, puberty, pornography and heterosexuality (Löfgren‐Mårtenson, 2012). For example, Bernert and Ogletree (2013) reported other discussion topics, such as love, dating, relationships, how to end relationships and for women in particular, sexual pleasure. Furthermore, Stoffelen et al. (2019) added the importance of accepting your own body, and the do's and don'ts of sex. There were several statements indicating that participants wanted sex‐related risks to be included in sex education, (e.g., unwanted pregnancies, STDs), as well as risk prevention (i.e., use of contraception, setting boundaries and asking for consent) (Löfgren‐Mårtenson, 2012; Schaafsma et al., 2017; Sitter et al., 2019).

Concerning the provision of sex education, the attitudes of participants with intellectual disabilities can roughly be divided into three features that contribute to successful sex education. First, a good student–teacher relation was cited as being important (Abbott & Burns, 2007; Löfgren‐Mårtenson, 2012; Schaafsma et al., 2017). Second, participants believed sex education should align with the specific educational needs of the individual student, in terms of difficulty, repetition and preferred educational method (Löfgren‐Mårtenson, 2012; Oakes & Thorpe, 2019). Third, the content should match with the maturity of the student, that is, not too young and not too old (Löfgren‐Mårtenson, 2012; Oakes & Thorpe, 2019). As one participant explained: ‘*Good to have sex education when you are 16–17 years old, because then it's actually about how to have sex and how you protect yourself from pregnancy and STDs*’ (Löfgren‐Mårtenson, 2012, p. 220).

### 3.6 Theme Six: Attitudes towards support provided by caregivers

In 27 studies, the attitudes of participants with intellectual disabilities towards the sexuality‐related support provided by family and support staff were identified, and concerned (1) experienced supportive roles of family and support staff, (2) the need for approval of sexual experiences and (3) sharing sexuality‐related questions with family and support staff.

First, participants with intellectual disabilities described their views about the supportive roles performed by family (*n* = 3) and support staff (*n* = 13). According to participants, family support was needed to invite participant's partners over, for example, for dinner (Turner & Crane, 2016b) or to help ‘*if you're going through a rough time*’ (Bane et al., 2012, p. 118). Similarly, support staff were viewed as ‘*necessary*’ (e.g., Rushbrooke et al., 2014, p. 538), albeit sometimes unavailable (e.g., ‘*They don't have time*’; Stoffelen et al., 2018, p. 254). Support staff were viewed by some as supportive, such as in the following account: ‘*She encouraged me to find a partner*’ (Neuman, 2020, p. 136). Others complained that support staff tried ‘*to embarrass [them] in front of [partner]*’ (Rushbrooke et al., 2014, p. 538). Some participants believed that support staff found it difficult to answer their questions, such as on dating (Kelly et al., 2009; Stoffelen et al., 2013). Moreover, participants believed support staff found LGBT‐related questions especially difficult (Abbott & Burns, 2007; Dinwoodie et al., 2016). As one participant illustrated: ‘*[W]hen you tell them I need support for this [gay sexuality] as well, then they don't want to support you with that bit*’ (Dinwoodie et al., 2016, p. 8).

Second, participants with intellectual disabilities expressed their belief that they needed approval from family, support staff and/or the service organisation. Regarding their family, the participants mostly felt that they needed permission for having a (sexual) relationship or getting married, and expected that this would not be allowed (e.g., Buljevac et al., 2020; Chou et al., 2015; Lesseliers & Hove, 2002; Rojas et al., 2016; Turner & Crane, 2016a; Wheeler, 2007). Support staff also were perceived by some as being restrictive, citing examples of being punished for their intimate relations (e.g., Buljevac et al., 2020). However, other participants felt that support staff were supportive of their intimate relationships (Fitzgerald & Withers, 2013; Lesseliers & Hove, 2002). Some participants believed that it was service policy and the law that prevented them from having relations (e.g., Abbott & Burns, 2007; Kelly et al., 2009; Lesseliers & Hove, 2002; Turner & Crane, 2016b). Finally, some participants believed that they should make their own decisions concerning their sexuality. For example: ‘*They shouldn't rule your life*’ (Kelly et al., 2009, p. 314), ‘*I feel very, that my relationship with [my wife], well that is my business and nobody else's*’ (Rushbrooke et al., 2014, p. 537) and ‘*[Sexuality is] none of their [staff] business*’ (Schaafsma et al., 2017, p. 30).

Lastly, participants expressed attitudes centred on the circumstances in which they would share their sexual feelings with family and support staff (*n* = 16). As a result of earlier negative life experiences, some participants believed that it was not safe to share their sexual feelings with family and support staff (Abbott & Burns, 2007; Healy et al., 2009), fearing they would experience discrimination (Abbott & Burns, 2007) or be ignored (Kelly et al., 2009; Stoffelen et al., 2013). As one participant illustrated: ‘…*. you'd be tryin' to explain to them, but they won't listen*’ (Kelly et al., 2009, p. 313). Furthermore, participants felt embarrassed when anticipating talking about sexuality with their family or support staff (Chou et al., 2008; Healy et al., 2009; Oakes & Thorpe, 2019; Schaafsma et al., 2017; Turner & Crane, 2016b). Although most participants felt reluctant to share their sexual feelings with family and support staff, some had, sometimes to their own surprise, a positive experience, which allowed them to elaborate further, and explore their sexuality: ‘*I told the social worker [about my relationship]. She was very open about it, says I can do what I like. I wasn't expecting her to be so nice, but then she is very nice*’ (Kelly et al., 2009, p. 312).

## 4 DISCUSSION

Motivated by the importance of sexual health for people with intellectual disabilities, the present systematic review aimed to provide an up‐to‐date overview of the attitudes as reported for people with mild and moderate intellectual disabilities towards their sexuality. In the analyses, six themes emerged, which together appeared to represent the broad conceptualization of sexuality of the WHO (2015). Themes concerned attitudes regarding: sexual behaviour, sexual identity, intimate relationships, barriers to sexual expression, sex education and support provided by caregivers. Integrating the results of this review leads to several implications for research and practice, which are further elaborated below.

### 4.1 Implications for research and practice

#### 4.1.1 A diversity in attitudes concerning sexuality

In the current review, a diverse set of attitudes towards sexuality, both restrictive and supportive, was identified. This review identified some studies in which people with intellectual disabilities: perceived sexual behaviour and intimate relationships as unpleasant, explicitly dismissed the notion of having a sexual relationship and/or seemed to have less favourable attitudes towards sexual behaviour (e.g., preferring not to wear condoms or wanting sex to play) (e.g., Bernert & Ogletree, 2013). However, this review also identified some studies that reported people with intellectual disabilities who indicated to enjoy (aspects of) sexual behaviour and relationships, and who appeared to take care when considering sexual relationships (i.e., being aware of the importance of sexual consent and sexual risks). Moreover, in relationships, some people with intellectual disabilities mostly desired to share and care for their partners, which can be considered a more comprehensive desire than having sexual contact. Also, there are examples of people with intellectual disabilities who realised that their own social skills could form a barrier towards intimate relationships and, therefore, valued support in finding and maintaining relationships. In summary, people with intellectual disabilities seem to hold a variety of attitudes towards sexuality. For professionals charged with the task of sexuality support, it might be worth considering to evaluate the attitudes of the individual service user, and assess the need for directed support and education. Future research is needed to determine the best forms of support for people with intellectual disabilities to express their sexuality‐related attitudes.

#### 4.1.2 Lack of focus on auto‐erotic behaviour

In the present review, little was reported on auto‐erotic behaviour (e.g., masturbation), and, when it was, it was often mentioned euphemistically by people with intellectual disabilities. This finding contradicts the prevailing view that auto‐erotic behaviour is the most common form of sexual behaviour for people with intellectual disabilities (Kaeser, 1996), and for some, the *only* way to express sexuality (Kijak, 2013; Rushbrooke et al., 2014). The low occurrence of reported attitudes concerning auto‐erotic behaviour might be indicative of a taboo culture (Kijak, 2011), possibly induced by support staff and relatives. For instance, some support staff and family caregivers view auto‐erotic behaviour as problematic, and, more specifically, as an early indication of future abusive behaviour (Cambridge et al., 2003; Kaeser, 1996). Consequently, some people with intellectual disabilities may have felt obstructed to speak freely about auto‐erotic expressions. Otherwise, these participants might have been unaware of the possibility of having auto‐erotic expressions. Either way, not talking or not knowing about auto‐erotic behaviour is unfortunate, because, auto‐erotic behaviour can have a number of benefits for people with intellectual disabilities, such as tension release, getting to know your own body and exploring one's sexual preferences (Cambridge et al., 2003; Kijak, 2011). Future research could further explore the attitudes of people with intellectual disabilities towards auto‐erotic behaviour, and, more specifically, whether or not people with intellectual disabilities acknowledge these benefits, which, in turn, would allow for better support and education.

#### 4.1.3 The role of sexuality support and education

In the current review, a significant part of the data represented references to sexuality support and education. Some participants with intellectual disabilities appeared to agree with the view that support and education have a profound influence on their ability for sexual expression (Servais, 2006; Wilkinson et al., 2015). The other way around, some participants noted that a lack of (appropriate) support and education could actually hinder one's ability to engage in sexual experiences. For instance, some people with intellectual disabilities either felt that their questions were ignored, that the answers were unsatisfactory, or that sharing their sexual feelings resulted in regulation or restriction. With their answers remaining unanswered, people with intellectual disabilities may opt to engage with opportunistic, less reliable sources of sex education (e.g., television, Internet), possibly resulting in incorrect knowledge, restrictive attitudes (Jahoda & Pownall, 2014; Strasburger et al., 2010) and engaging in forms of sexual behaviour that carried an increased sexual risk (i.e., having sex in public places, engaging in sexual interactions without the knowledge of family and support staff and having unprotected sexual intercourse).

The provision of high‐quality sex education thus seems to be crucial. However, as some participants identified relevant subjects for sex education, most of these subjects appear to focus on rather technical aspects of sexuality, for instance the biology, how to have sex, how to have children and how to date. In addition to these technical aspects, sexual pleasure might be relevant for the future development of sexuality support and sex education (Ford et al., 2019). Some people with intellectual disabilities did mention the topic of sexual pleasure to be important, especially for women (Bernert & Ogletree, 2013). Besides the relevant subjects, participants identified key features of sexuality support and education. For example, some participants referred to the importance of attuning such initiatives to the individual by appealing to their preferred learning style (Dukes & McGuire, 2009), their specific communication and social skills, their past experiences, level of maturity and their needs and desires. If the quality of sexuality‐related support and education would be improved, people with intellectual disabilities might be more motivated to share their sexuality‐related experiences and attitudes, and might be more open to receive sexuality‐related support (Harden, 2014; Williams et al., 2013). Further research on the improvement of sexuality support and sex education is recommended to further explore its potential impact on sexual health for people with intellectual disabilities.

### 4.2 Limitations

The present study primarily had one limitation. The interpretation of the participants' quotes was sometimes complicated by identifying references towards generic expressions like ‘sexuality’ or ‘sex’. In such cases, it was not always clear what aspect of sexuality was being referred to. The participants' use of generic expressions might be indicative of a lack of sexual knowledge (Kijak, 2013; Schaafsma et al., 2017; Siebelink et al., 2006), or of feelings of embarrassment in talking about sexuality (Healy et al., 2009). However, as illustrated above, some participants with intellectual disabilities were more than capable of expressing themselves via a wide range of terms, adding details and sincerity to their expressions. Furthermore, some participants provided specific details when describing their sexual desires. When people with intellectual disabilities are only capable of expressing their sexual feelings in general terms, it can be a challenge for professionals and family to attune their support and education to the individuals' needs (Schaafsma et al., 2017). Particularly in these cases, professionals and support staff might want to consider the use of assisted communication to compensate for impossibilities in verbal differentiation (Werner, 2012).

## 5 CONCLUSION

People with intellectual disabilities proof to be capable of presenting a wide variety of attitudes regarding the broad concept of sexuality. Overall, the findings suggest that people with intellectual disabilities have heterogeneous desires regarding sexuality. Through the use of assisted communication, people with intellectual disabilities seem to be able to express their sexual desires, needs and attitudes, which, in turn, allows for the improvement of individual support and education. The current review provides concrete starting points for future research and suggests new initiatives and perspectives in clinical practice in order to contribute to the sexual health of people with intellectual disabilities.

## CONFLICT OF INTEREST

We have no known conflict of interest to disclose.

## Data Availability

The data that support the findings of this study are available from the corresponding author upon reasonable request.
